# Supplementary material for: Dynamic transcriptomic profiles of zebrafish gills in response to zinc supplementation
Source: BMC Genomics. 2010 Oct 11;11:553. doi: 10.1186/1471-2164-11-553 (PMC3091702; doi:10.1186/1471-2164-11-553)
Supplement: Additional file 2 — Interactive Direct Interaction Network representing the molecular interactions between zinc, copper, iron, calcium and proteins encoded by transcripts changed by zinc supplementation. Mini web-site containing index.html and hyperlinked pages in subdirectory describing a Direct Interaction Network automatically generated based on curated interactions contained within the proprietary PathwayArchitect database. Ovals represent proteins and the circles symbolize metal ions. Objects are coloured by their abundance in zebrafish at the time-point they were significantly different from the control is a scale from -4 fold (dark green) to +4 fold (dark red). Where significant differences were found at more than one time-point, the colour overlay shows expression at the first instance. Dark blue squares denote 'binding', and light blue squares 'expression'; green squares stand for 'regulation', green diamonds for 'metabolism', and green circles for 'promoter binding'. Arrow heads indicate directionality of the interaction where annotated. All nodes and edges can be further interrogated by selecting the relative area of the image. [file 1471-2164-11-553-S2.zip › PathwayArchitect Zn xs DIN/82573.html]

# PROTEIN: BMP7

|  |  |
| --- | --- |
| Name | BMP7 |
| Type | PROTEIN |
| Description | bone morphogenetic protein 7 (osteogenic protein 1) |
| Note | The bone morphogenetic proteins (BMPs) are a family of secreted signaling molecules that can induce ectopic bone growth. Many BMPs are part of the transforming growth factor-beta (TGFB) superfamily. BMPs were originally identified by an ability of demineralized bone extract to induce endochondral osteogenesis in vivo in an extraskeletal site. Based on its expression early in embryogenesis, the BMP encoded by this gene has a proposed role in early development. In addition, the fact that this BMP is closely related to BMP5 and BMP7 has lead to speculation of possible bone inductive activity. |
| Alias | Eptotermin alfa |
|  | Bmp7 |
|  | bone morphogenetic protein 7 |
|  | OP1 |
|  | Op1 |
|  | osteogenic protein 1 |
|  | BMP-7 |
|  | bone moorphogenic protein-7 |
|  | Bmp-7 |
|  | Osteogenic protein 1 |
|  | BMP7 |
|  | OP-1 |


---

|  |  |
| --- | --- |
| GO Component | extracellular space |


---

|  |  |
| --- | --- |
| GO ID | GO:0005615 |
|  | GO:0008083 |
|  | GO:0042325 |
|  | GO:0051216 |
|  | GO:0001503 |
|  | GO:0035239 |
|  | GO:0001763 |
|  | GO:0007389 |
|  | GO:0001707 |
|  | GO:0005515 |
|  | GO:0008201 |
|  | GO:0048468 |
|  | GO:0007411 |
|  | GO:0045597 |
|  | GO:0030154 |
|  | GO:0005125 |
|  | GO:0030509 |
|  | GO:0042475 |
|  | GO:0009887 |
|  | GO:0007435 |
|  | GO:0007275 |
|  | GO:0040007 |


---

|  |  |
| --- | --- |
| MIM | MIM:112267 |


---

|  |  |
| --- | --- |
| Connectivity | 470 |


---

|  |  |
| --- | --- |
| Entrez ID | 85272 |
|  | 655 |
|  | 12162 |


---

|  |  |
| --- | --- |
| Agilent ID | A\_51\_P492366 |
|  | A\_23\_P68487 |
|  | A\_53\_P125398 |
|  | A\_43\_P15871 |
|  | A\_24\_P91566 |
|  | A\_52\_P278354 |
|  | A\_14\_P110420 |
|  | A\_44\_P449806 |
|  | A\_23\_P154643 |
|  | A\_14\_P118519 |
|  | A\_23\_P68488 |
|  | A\_53\_P169777 |
|  | A\_14\_P100351 |
|  | A\_53\_P115874 |


---

|  |  |
| --- | --- |
| Cellular Localization | Extracellular region |


---

|  |  |
| --- | --- |
| DbXref | KEGG pathway##04340##Hedgehog signaling pathway##http://www.genome.jp/dbget-bin/show\_pathway?mmu04340+12162 |
|  | KEGG pathway##04340##Hedgehog signaling pathway##http://www.genome.jp/dbget-bin/show\_pathway?hsa04340+655 |
|  | KEGG pathway##04060##Cytokine-cytokine receptor interaction##http://www.genome.jp/dbget-bin/show\_pathway?hsa04060+655 |
|  | KEGG pathway##04350##TGF-beta signaling pathway##http://www.genome.jp/dbget-bin/show\_pathway?hsa04350+655 |
|  | KEGG pathway##04060##Cytokine-cytokine receptor interaction##http://www.genome.jp/dbget-bin/show\_pathway?mmu04060+12162 |
|  | KEGG pathway##04350##TGF-beta signaling pathway##http://www.genome.jp/dbget-bin/show\_pathway?mmu04350+12162 |
|  | KEGG pathway##04350##TGF-beta signaling pathway##http://www.genome.jp/dbget-bin/show\_pathway?rno04350+85272 |
|  | KEGG pathway##04340##Hedgehog signaling pathway##http://www.genome.jp/dbget-bin/show\_pathway?rno04340+85272 |


---

|  |  |
| --- | --- |
| Pathway | Zn xs inventory |
|  | Zn xs DIN |


---

|  |  |
| --- | --- |
| GO Process | cell differentiation |
|  | tube morphogenesis |
|  | branching morphogenesis |
|  | regulation of phosphorylation |
|  | positive regulation of cell differentiation |
|  | growth |
|  | organ morphogenesis |
|  | ossification |
|  | odontogenesis (sensu Vertebrata) |
|  | axon guidance |
|  | pattern specification |
|  | BMP signaling pathway |
|  | cell development |
|  | salivary gland morphogenesis |
|  | development |
|  | cartilage development |
|  | mesoderm formation |


---

|  |  |
| --- | --- |
| UniGene | Rn.18030 |
|  | Hs.473163 |
|  | Mm.595 |


---

|  |  |
| --- | --- |
| Affymetrix Probeset ID | 109325\_at |
|  | 1389403\_at |
|  | 1418910\_at |
|  | 1432410\_a\_at |
|  | 209590\_at |
|  | 209591\_s\_at |
|  | 211259\_3p\_s\_at |
|  | 211259\_s\_at |
|  | 211260\_at |
|  | 38515\_at |
|  | 49267\_at |
|  | 49273\_g\_at |
|  | 64137\_at |
|  | 93243\_at |
|  | D29769\_at |
|  | g13279013\_3p\_a\_at |
|  | g13279013\_3p\_at |
|  | g13279013\_3p\_x\_at |
|  | g339563\_3p\_a\_at |
|  | Hs.170195.1.A2\_3p\_at |
|  | rc\_AI013715\_at |
|  | X51801\_at |
|  | X56906\_s\_at |
|  | RC\_AA029597\_at |
|  | RC\_AA598920\_at |


---

|  |  |
| --- | --- |
| GO Function | protein binding |
|  | growth factor activity |
|  | heparin binding |
|  | cytokine activity |


---

|  |  |
| --- | --- |
| Nucleotide | NM\_001719 |
|  | CQ786476 |
|  | AF210054 |
|  | D29769 |
|  | BC008584 |
|  | AL157414 |
|  | BC010771 |
|  | X56906 |
|  | NM\_007557 |
|  | AF100787 |
|  | AF289090 |
|  | AL122058 |
|  | BC004248 |
|  | AK020411 |
|  | M60316 |
|  | XM\_342591 |
|  | X51801 |


---

|  |  |
| --- | --- |
| Protein | BAA31853 |
|  | NP\_031583 |
|  | AAD27804 |
|  | P18075 |
|  | NP\_001710 |
|  | CAA40222 |
|  | AAH08584 |
|  | CAC08434 |
|  | AAF97256 |
|  | XP\_342592 |
|  | CAA36100 |
|  | AAG43508 |
|  | AAA36738 |
|  | AAH04248 |
|  | CAB90273 |
|  | AAH10771 |
|  | CAG17752 |
|  | P23359 |


---

|  |  |
| --- | --- |
| Organism | Mammal |


---

|  |  |
| --- | --- |
| Location | chromosome 20, 20q13 (Homo sapiens) |
|  | chromosome 3, 3q42 (Rattus norvegicus) |
|  | 2 102.0 cM (Mus musculus) |
|  | chromosome 2, 2 102.0 cM, 2 H3 (Mus musculus) |


---

|  |  |
| --- | --- |
